# Repetitive Transcranial Magnetic Stimulation for Spasticity in Stroke and Other Neuromotor Disorders: A Systematic Review of Randomized Clinical Trials

**DOI:** 10.3390/jcm15051932

**Published:** 2026-03-04

**Authors:** Michele Iacona, Rosario Ferlito, Rita Bella, Mariagiovanna Cantone, Raffaele Ferri, Francesco Fisicaro, Salvatore Giunta, Pietro Marano, Maria P. Mogavero, Vito Pavone, Manuela Pennisi, Gianluca Testa, Davide N. Tringali, Giuseppe Lanza

**Affiliations:** 1 Department of Medical and Surgical Sciences and Advanced Technologies “G.F. Ingrassia”, University of Catania, 95123 Catania, Italy; m.iacona26@gmail.com (M.I.); rbella@unict.it (R.B.); davide.tringali02@icloud.com (D.N.T.); 2 Department of Biomedical and Biotechnological Sciences, University of Catania, 95123 Catania, Italy; ferlito.rosario@libero.it (R.F.); sgiunta@unict.it (S.G.); manuela.pennisi@unict.it (M.P.); 3 Neurology Unit, Policlinico University Hospital “G. Rodolico-San Marco”, 95123 Catania, Italy; m.cantone@policlinico.unict.it; 4 Oasi Research Institute-IRCCS, 94018 Troina, Italy; rferri@oasi.en.it (R.F.); pmarano@oasi.en.it (P.M.); paola_mogavero@libero.it (M.P.M.); 5 Primary Health Care Unit, Provincial Health Authority of Siracusa, 96100 Siracusa, Italy; 6 Department of Surgery and Medical-Surgical Specialties, University of Catania, 95123 Catania, Italy; vpavone@unict.it (V.P.); gianluca.testa@unict.it (G.T.)

**Keywords:** spasticity, repetitive transcranial magnetic stimulation, neuromodulation, neurorehabilitation, neural plasticity, stroke

## Abstract

**Background**: Spasticity is a common and disabling feature of several neuromotor disorders. Repetitive transcranial magnetic stimulation (rTMS) has been proposed as a non-invasive approach to modulate corticospinal excitability and reduce spasticity, although its clinical effectiveness remains debated. This systematic review evaluated the efficacy and safety of rTMS in reducing spasticity in stroke and other neuromotor conditions. **Methods**: A systematic search of PubMed, Scopus, and Cochrane Library was conducted up to June 2025 in accordance with PRISMA 2020 guidelines. Eligible studies were randomized controlled trials (RCTs) comparing rTMS with sham stimulation or conventional therapy and assessing spasticity using validated scales, primarily the Modified Ashworth Scale. Included populations comprised patients with stroke, spinal cord injury, multiple sclerosis, cerebral palsy, and hereditary spastic paraplegia. Risk of bias was assessed using the RoB 2.0 tool, and certainty of evidence was evaluated with GRADE. **Results**: Twenty-six RCTs were included, mainly involving stroke patients. Most studies reported a significant reduction in spasticity with rTMS compared with control interventions. Low-frequency stimulation was commonly used after stroke, while excitatory protocols predominated in other conditions. Benefits generally persisted for up to 12 weeks. Evidence quality was moderate, and no serious adverse events were reported. **Conclusions**: rTMS appears to be a safe and promising adjunctive treatment for spasticity across neuromotor disorders. However, protocol heterogeneity and small sample sizes limit definitive clinical recommendations, highlighting the need for standardized, larger-scale studies.

## 1. Introduction

Spasticity is a common clinical manifestation of many neuromotor disorders affecting the nervous system, including stroke, multiple sclerosis, traumatic spinal cord injury, cerebral palsy, and some neurodegenerative disorders (such as hereditary spastic paraplegia). Spasticity consists of an increase in muscle tone, mainly involving antigravity muscles, i.e., flexors of the upper limbs and extensors of the lower limbs [1]. Assessment of spasticity is usually performed by the examiner through passive mobilization, investigating the involuntary resistance that the limb offers to movement, which increases in proportion to the speed of stretch. Spasticity can significantly impair motor activity, functional independence, and quality of life, as well as increasing the care burden and healthcare costs [2,3,4].

To date, spasticity management relies on pharmacological treatments, rehabilitative approaches, and, in selected cases, surgical interventions. However, these strategies present several limitations: antispastic drugs may cause insidious side effects, such as sedation, muscle weakness, or long-term tolerance; physical therapy requires intensive and continuous programs, often yielding modest results, especially in cases of severe spasticity; finally, surgical interventions are invasive and reserved for refractory conditions only. These critical issues prompt the search for innovative, effective, and less invasive therapeutic options [5].

In recent years, there has been growing interest in non-invasive neuromodulatory techniques, including repetitive transcranial magnetic stimulation (rTMS), as a possible alternative or adjunctive therapeutic option [6]. Briefly, TMS is a non-invasive brain stimulation method acting on the cerebral cortex through the administration of magnetic pulses directly applied to the scalp [7,8,9,10]. Based on single-pulse TMS, therapeutic protocols such as rTMS have been developed; this consists of delivering trains of pulses, at a preset frequency, capable of inducing long-term changes in cortical excitability and synaptic plasticity [5]. Repetitive TMS can exert inhibitory or facilitatory effects on cortical activity, depending on the frequency and stimulation pattern used, with low-frequency (LF, ≤1 Hz) usually inhibitory and high-frequency (HF, >5 Hz) usually excitatory. Among different protocols, the most innovative, i.e., the continuous theta burst stimulation (cTBS) and intermittent TBS (iTBS), mimics physiological neuronal firing rhythms and allows for the induction of neuroplastic effects with short stimulation durations [11], also allowing to monitor cortical excitability and synaptic plasticity over time [12].

In this context, increasing attention has been directed toward rehabilitative and neuromodulatory interventions as part of a multimodal approach to spasticity management. Beyond conventional physiotherapy, several neuromodulatory techniques have been explored, including rTMS, transcranial direct current stimulation, peripheral nerve stimulation, robotic-assisted training, and virtual reality-based rehabilitation. These approaches aim to promote adaptive neuroplasticity and enhance motor relearning through targeted modulation of cortical and subcortical networks. Moreover, emerging evidence suggests that combining neuromodulatory interventions with pharmacological treatments and task-oriented rehabilitation may produce synergistic effects, potentially maximizing functional recovery and long-term symptom control [13]. This integrative perspective fosters multidisciplinary collaboration among neurologists, rehabilitation physicians, physiotherapists, engineers, and neuroscientists, and represents a promising and evolving field in neurorehabilitation research.

Regarding spasticity, substantial evidence suggests a potential therapeutic use for rTMS in these patients, although data are heterogeneous regarding protocols, populations, and outcomes considered, making a definitive synthesis of results complex to reach yet. Additionally, available evidence remains fragmented across diagnoses and highly variable stimulation parameters, and an updated synthesis focused specifically on randomized clinical trials (RCTs) and structured certainty assessment is warranted. The high socioeconomic impact of spasticity and the increasing prevalence of neuromotor disorders, indeed, underscore the urgency of identifying more effective, safe, and sustainable therapeutic strategies. In this context, although this review also includes neuromotor conditions other than stroke, it should be acknowledged that post-stroke spasticity represents the largest and clinically most relevant subgroup in the available randomized evidence.

Based on this background, the present systematic review aims to evaluate the safety and efficacy of rTMS in reducing spasticity in subjects with different neuromotor disorders, by analysing all the RCTs published in the relevant databases. As such, a systematic review was conducted in accordance with the Preferred Reporting Items for Systematic Reviews and Meta-Analyses (PRISMA) guidelines to ensure transparency, reproducibility, and methodological rigor [14]. The objective of the present review is to provide an updated contribution to the role of rTMS in the rehabilitative management of spasticity.

## 2. Materials and Methods

This systematic review was conducted in accordance with the PRISMA 2020 and the completed PRISMA checklist provided as Supplementary Materials. The selection of studies took place in two stages: in the first stage, two independent reviewers (M.I. and D.N.T.) examined the titles and abstracts of the articles identified through database searches; in the second stage, the selected texts were read in full to verify their adherence to the pre-established inclusion criteria. Any discrepancies between the reviewers were resolved through discussion and, if no consensus was reached, through the decision of a third author (G.L.). The selection process was documented using a PRISMA flow chart (Supplementary Materials [15]). Only RCTs that evaluated the effect of rTMS on spasticity in patients with neuromotor disorders were included.

Inclusion criteria were the diagnosis of spasticity; intervention with rTMS; outcome measured with standardised scales, such as the modified Ashworth Scale (MAS), the Tardieu Scale, or other validated clinical scales for spasticity; RCTs; any other relevant study retrieved from the reference list of the studies included in the review. On the contrary, the following have been excluded: animal studies; studies on disorders not related to spasticity; case reports, case series, editorials, commentaries, etc.; abstracts with missing or incomplete text; full text not available/not accessible; non-English written articles.

The search and selection of studies was guided by the following PICO model:

- Population (P): patients with spasticity secondary to neuromotor disorders, including stroke, multiple sclerosis, cerebral palsy, spinal cord injury, head trauma, amyotrophic lateral sclerosis, and other neurological conditions causing spasticity;
- Intervention (I): rTMS administered using any protocol (HF, LF, TBS), with variable treatment duration and different brain targets, e.g., primary motor cortex (M1), premotor cortex, lesioned/non-lesioned hemisphere;
- Comparison (C): sham (fictitious) stimulation, placebo, or other standard therapies for spasticity, including pharmacological treatments (e.g., tizanidine, baclofen, botulinum toxin, etc.) and physiotherapy;
- Outcome (O): change in spasticity assessed using standardised scales, such as MAS, Tardieu Scale, or other validated scales for spasticity.

A systematic search was conducted in three major international databases: PubMed, Scopus, and Cochrane Library, from database inception to June 2025. Eligible articles present in the databases during that period were included, using the following PubMed search strings: (“repetitive transcranial magnetic stimulation” OR “rTMS”) AND (“spasticity” OR “muscle spasticity”). This search produced 132 identified articles; after reading both title and abstract, 24 were selected, one of which was excluded after full-text analysis. The following search string was used on Scopus: TITLE-ABS-KEY ((“repetitive transcranial magnetic stimulation” OR “rTMS”) AND (“spasticity” OR “muscle spasticity”)) AND (LIMIT-TO (DOCTYPE, “ar”)). A total of 122 articles were identified; after screening for duplicates and reading the remaining full texts, three articles were included. On Cochrane Library (London, UK), the search string used was: (“repetitive transcranial magnetic stimulation” OR “rTMS” AND “spasticity” OR “muscle spasticity” AND “randomized controlled trial”). This string returned 25 identified articles; of these, four were selected after initial screening and duplicate removal, but then excluded after in-depth analysis. All results were exported and managed using Excel for search, screening, selection, and removal of duplicates.

The following data were extracted for each study included: Author, Year, Population Pathology, Study Type, Sample Size, Intervention (rTMS), Control, Outcome (scales), Follow-up Duration, Main Results. The risk of bias (RoB) for each study was assessed using the RoB 2.0 (Revised Cochrane Risk of Bias tool) [16], which considers five main domains: bias in randomisation; bias due to deviations from the assigned intervention; bias in outcome measurement; bias in handling missing data; bias in selection of reported results. Finally, to interpret the overall strength of the evidence, a structured assessment of the methodological quality of the studies and the level of certainty of the outcomes was conducted using the GRADE (Grading of Recommendations, Assessment, Development and Evaluation) system [17], which considers 5 main domains: risk of bias, inconsistency, imprecision, indirectness, and risk of publication bias. Based on these criteria, the certainty of the evidence was classified into four levels: high, moderate, low, or very low.

## 3. Results

### 3.1. Preliminary Considerations

**Figure 1:**
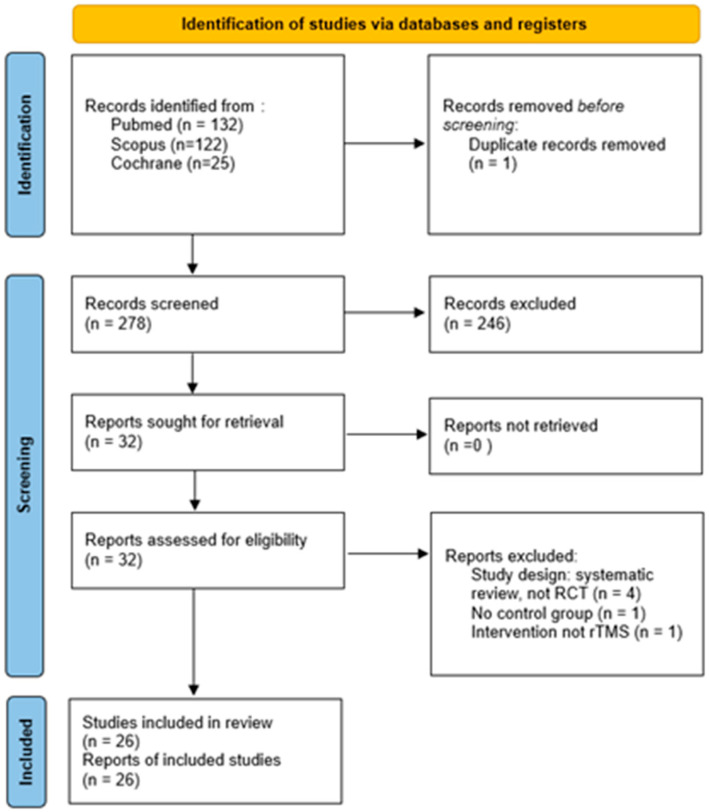
PRISMA 2020 flow diagram of the studies retrieved, screened, selected, and eventually included (for further details on the PRISMA checklist, please see the Supplementary Materials).

The multi-database systematic search identified a total of 279 articles: 132 from PubMed, 122 from Scopus, and 25 from the Cochrane Library. After removing duplicates, 246 were further excluded based on their titles and abstracts. The remaining 32 articles were evaluated by accurately reading each full text; six of these were excluded for reasons related to the study design or irrelevant intervention. After this process, 26 RCTs were eventually included in the review. The selection process is illustrated in the PRISMA diagram shown in Figure 1, whereas Table 1 presents the summary of results.

It is important to clarify that, in all the studies included, the primary outcome was spasticity measured using validated scales (mainly the MAS, the Tardieu Scale, or similar), while the other indicators reported (i.e., motor function, walking, pain, quality of life and neurophysiological measures) represent secondary outcomes, which were assessed when available.

**Figure 2:**
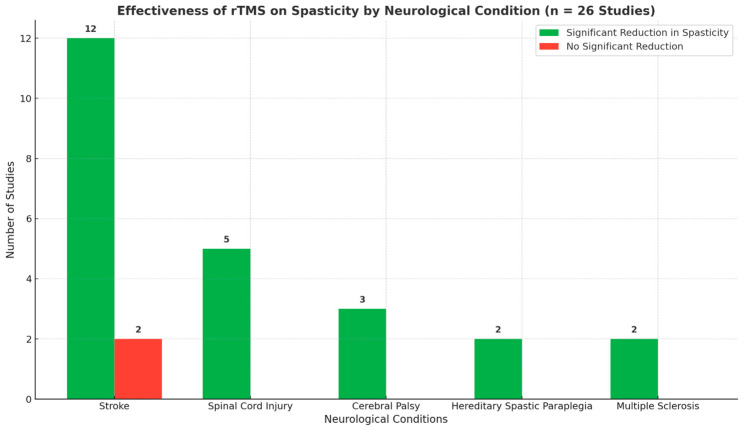
Number of studies reporting a significant reduction in spasticity following repetitive transcranial magnetic stimulation (rTMS), grouped by the neuromotor condition considered in the present review.

Due to the high clinical heterogeneity (mostly due to different pathologies or stimulation protocols) and methodological heterogeneity (different outcome scales and timing), a meta-analysis of the results could not be conducted. A quantitative analysis, indeed, requires substantial clinical and methodological homogeneity in terms of diagnoses, targets, frequencies, session number, co-interventions, and outcome timing. Therefore, findings are presented as a global synthesis grouped by condition. For the sake of clarity, it was useful to group the 26 studies selected by pathology (i.e., stroke, spinal cord injury, cerebral palsy, multiple sclerosis, hereditary spastic paraplegia) and compare the rTMS protocols used, the outcome measures considered, and the results obtained (Figure 2).

For the same reasons, i.e., the clinical and methodological heterogeneity among the included trials, subgroup or sensitivity analyses were not performed. Although preliminary stratification by pathology was undertaken, further quantitative subgroup analyses based, e.g., on stimulation frequency, cortical target, coil type, treatment duration, or outcome timing, were not feasible because of the limited number of studies within each category here considered and the wide variability of protocols used across the studies. Similarly, sensitivity analyses excluding studies with higher risk of bias or crossover designs were not conducted, as such restrictions would have resulted in very small and underpowered subsets. Consequently, a narrative synthesis grouped by condition and protocol characteristics was considered the most appropriate approach.

### 3.2. Primary Outcome: Spasticity

We found 14 studies on stroke: most of these enrolled small samples (14–60 subjects) with spasticity of the upper or lower limbs at least 1–3 months after the ischaemic event. Inhibitory protocols (1 Hz or 0.5 Hz) applied to the contralateral (healthy motor) hemisphere for 5–20 sessions were mainly used; some studies, however, used 5 Hz on the affected hemisphere. All studies measured spasticity using the MAS or its variants. Studies with LF-rTMS on the healthy hemisphere showed a significant reduction in spasticity at the end of treatment, while no benefit was observed with sham treatment. For instance, in the study by Gottlieb et al. [18], it was observed that 1 Hz rTMS significantly reduced MAS scores. Barros Galvão et al. [28] found that 90% of patients treated with rTMS + physiotherapy reduced their MAS score by ≥1 point compared to 55% of the physiotherapy group only. Regarding safety, rTMS was well tolerated; the most common side effects reported were mild headache or transient skin discomfort, whereas no study reported any serious adverse effect.

Looking at evidence on spinal cord injury, five studies were analysed. Most of them were carried out on small group of patients (10–34 subjects) with incomplete spinal cord injury and used excitatory protocols with double cone coils on the motor representation of the legs (20 Hz) for 5–25 sessions, or other excitatory protocols (iTBS). For instance, the randomised crossover study by Kumru et al. [22] showed that, compared to the sham group, five sessions of real rTMS at 20 Hz significantly reduced lower limb spasticity as measured by MAS, with the effect persisting for one week. Interestingly, patients also complained less spasms and improved sleep. Also in this case, rTMS was found to be safe; some patients reported transient facial twitching, without any other manifestations, as well as no serious adverse effect.

The three studies involving children with spastic cerebral palsy used 10 Hz rTMS applied to the contralateral M1 for 20–40 sessions, always in combination with physiotherapy. Overall, the results of these studies showed a reduction in spasticity measured through the modified MAS; in particular, all lower limb muscles, except the gastrocnemius, showed a significant decrease in the score after 40 sessions. No adverse effect was reported and all the children involved completed all sessions.

Two studies were identified for multiple sclerosis, which included patients with a progressive form of the disease and adductor spasticity or paraparesis. HF-rTMS (20 Hz) or iTBS were compared with sham: in both studies, a significant reduction in spasticity was observed, while the control group showed no change. Interestingly, iTBS maintained the reduction in spasticity for at least 12 weeks, whereas rTMS also reduced pain and fatigue.

Finally, two studies were identified for hereditary spastic paraplegia. In the study by Antczak, J et al. [32], HF-rTMS (5 Hz) was applied for two weeks in these patients; the authors reported a long-lasting improvement in muscle strength and a decrease in proximal muscle spasticity (mean MAS score decreased from 4.96 to 3.29) but no improvement in the sham group. Although the procedure was generally safe, a single participant with hereditary spastic paraplegia involved in this trial experienced an epileptic seizure.

Lastly, for each of the included studies, a RoB assessment was performed using RoB 2.0 specifically developed for RCTs. The assessment for each domain was classified as low risk, some concerns, or high risk of bias. Based on these, an overall assessment was made for each study, and the summary of the results is shown in Table 2. To facilitate interpretation of the certainty of evidence for the main outcomes, the results of the GRADE assessment for key clinical endpoints are summarized in Table 3. Based on the GRADE assessment, the certainty of evidence supporting rTMS efficacy in reducing upper and lower limb spasticity was judged to be moderate. This rating reflects generally low risk of bias but was downgraded for inconsistency and imprecision related to protocol heterogeneity and limited sample sizes. Evidence regarding safety was also judged to be moderate, owing to the low incidence of serious adverse events but incomplete reporting in some trials. In contrast, evidence for secondary outcomes, including motor function and quality of life, was rated as low to very low, primarily because of heterogeneous outcome measures, small samples, inconsistent findings, and limited statistical power.

### 3.3. Secondary Outcomes: Motor Function, Gait, Pain, and Quality of Life

In addition to spasticity, several included studies also evaluated secondary outcomes, which are related to motor performance, functional independence, gait, pain, fatigue, and quality of life [18,23,25,26,28,30,31,32,34,36,37,38,39,40,42,43].

With regard to motor function, improvements were reported in multiple stroke and spinal cord injury trials using validated scales such as the Fugl–Meyer Assessment, Motricity Index, Action Research Arm Test, and Upper and Lower Extremity Motor Scores [18,23,25,26,27,36,38,40,42]. Significant gains in motor performance were observed in studies combining rTMS with rehabilitation or facilitation exercises, particularly in chronic and subacute stroke populations [23,25,26,28,38]. However, some trials did not demonstrate significant between-group differences, despite within-group improvements [27,33,35].

Functional independence and activities of daily living were assessed in several studies using instruments such as the Functional Independence Measure, Modified Barthel Index, and Motor Activity Log [25,26,28,31,40]. Moderate improvements in functional autonomy were reported in some stroke studies, particularly when rTMS was combined with physiotherapy or task-oriented training [25,26,28,40]. Nevertheless, these findings were inconsistent across trials, and several studies failed to show significant advantages over sham stimulation [31,33,35].

Gait and mobility outcomes were mainly evaluated in patients with stroke, spinal cord injury, and cerebral palsy using measures such as the Timed Up and Go test, 10-Meter Walking Test, gait analysis systems, and range-of-motion assessments [20,23,27,32,35,43]. Some studies reported improvements in walking speed, stride length, and lower-limb motor function, especially in cerebral palsy and incomplete spinal cord injury [20,32,37,43]. In contrast, other trials found no significant changes in gait parameters, suggesting variable responsiveness depending on disease stage, stimulation protocol, and rehabilitation context [27,35].

Pain, fatigue, and patient-reported symptoms were assessed primarily in studies involving multiple sclerosis and spinal cord injury [22,24,30,34]. Reductions in pain intensity and fatigue scores were observed in some trials using high-frequency rTMS or iTBS, particularly in patients with secondary progressive multiple sclerosis [24,30]. However, these effects were not consistently reproduced across studies and were often transient [22,34].

Finally, quality of life was evaluated in a limited number of trials using instruments such as the Stroke-Specific Quality of Life scale and related questionnaires [28,36,38]. While some studies reported modest improvements following combined neuromodulation and rehabilitation [28,38], most trials did not demonstrate statistically significant or clinically meaningful changes compared with control interventions [36].

Overall, although several studies reported favorable trends in motor function, functional independence, gait, and symptom burden, results for secondary outcomes were heterogeneous and less consistent than those observed for spasticity [18,23,24,25,26,27,28,30,31,32,34,36,37,38,39,40,42,43]. Improvements were more frequently reported in studies employing multi-session stimulation protocols and combined rehabilitation strategies [23,25,26,28,38,43]. However, small sample sizes, variable outcome instruments, and limited follow-up periods restricted the strength and generalizability of these findings.

## 4. Discussion

### 4.1. Main Findings

The main finding of this systematic review suggests that rTMS may be a promising therapeutic option for reducing spasticity across different neuromotor disorders, including stroke, spinal cord injury, multiple sclerosis, cerebral palsy, and hereditary spastic paraplegia. Most studies, especially on stroke and spinal cord injury, report a clinically significant reduction in spasticity according to MAS or related scales. In particular, rTMS appears to be most effective when applied daily for more than 10 sessions and when combined with physiotherapy or intensive training. The effects can last from 1 week to 12 weeks, with a trend to decrease after discontinuation. In stroke patients, LF (1 Hz, inhibitory) stimulation is preferred on the unaffected hemisphere to reduce interhemispheric cortical hyperactivity, while in other conditions, excitatory frequencies (5–20 Hz) are used on the affected motor cortex. In spinal cord injury, 20 Hz-rTMS or iTBS on the leg representation were those that improved spasticity the most. Regarding safety, rTMS was generally safe; rare side effects reported (headache, skin discomfort, facial twitching) were of mild entity and short duration, without any serious adverse event.

Most of the included studies reported a significant reduction in spasticity, assessed mainly using the MAS, compared with sham stimulation or conventional therapies alone. However, there was considerable heterogeneity in rTMS protocols applied, cortical targets stimulated, and outcome scales used, making it difficult to generalise the results unequivocally. Additionally, safety appears favourable when rTMS was delivered within established safety recommendations and after appropriate screening for seizure risk factors, although isolated events highlight the need for standardized reporting.

When compared with the broader literature, the pattern emerging from our synthesis is consistent with pooled evidence indicating moderate reductions in MAS, particularly for upper-limb spasticity after stroke and when stimulation is delivered in multi-session courses (often >10 sessions) and combined with rehabilitation [44]. However, we also emphasize substantial protocol heterogeneity (frequency, intensity, target, coil type, and co-interventions) and variable durability, which likely reflects both biological differences across aetiologies and methodological differences in spasticity measurement. In particular, the results observed in patients with stroke and spinal cord injuries confirm the potential of rTMS as an adjunctive treatment to standard rehabilitation programmes, while for other non-stroke conditions, such as multiple sclerosis and hereditary spastic paraplegia, the evidence is still preliminary and based on small sample sizes [45].

The apparent divergence in stimulation strategies across conditions is biologically plausible. In stroke, contralesional inhibitory protocols (e.g., 1 Hz) may reduce maladaptive interhemispheric inhibition and rebalance cortical excitability, indirectly mitigating spasticity-related hyperexcitability. Conversely, in spinal cord injury and other non-stroke conditions, excitatory paradigms HF-rTMS or iTBS) may aim to enhance residual cortico-spinal drive and facilitate neuroplastic reorganization of the descending contralateral motor pathways [46,47]. Although the exact mechanisms remain incompletely defined, several trials reporting spasticity reduction also describe changes in cortical and/or spinal excitability measures, supporting a clear neuromodulatory contribution beyond a potential contribution of a “placebo effect” [48,49].

Neurobiologically, it is intriguing to face these findings with the pathophysiological basis underlying spasticity. As known, spasticity is increasingly viewed as a multi-level phenomenon in which hyperexcitability of the stretch reflex emerges from maladaptive reweighting of descending motor control rather than from a pure “spinal” disorder. After upper motor neuron injury, loss of cortico-spinal and other supraspinal inputs reduces tonic inhibition into spinal interneuronal circuits, while parallel upregulation of brainstem pathways (particularly reticulospinal projections) can amplify segmental reflex gain and muscle co-contraction. This framework helps explain why clinical spasticity correlates only partially with weakness and why it may evolve over time after the lesion [4,50]. In this context, the clinical utility of rTMS may lie in its ability to engage plasticity across several nodes of the so called “spasticity network”. At the cortical level, indeed, repeated trains of stimulation can induce long-term potentiation-/long term depression-like changes in synaptic efficacy and shift the excitation–inhibition balance within M1 and connected premotor areas. In stroke, in particular, inhibitory stimulation of the contralesional M1 is hypothesized to dampen excessive transcallosal inhibition and restore a more physiological interhemispheric balance, thereby improving voluntary drive and reducing the involuntary “overflow” that contributes to spastic posturing. The predominance of contralesional 1-Hz protocols in post-stroke RCTs is therefore mechanistically coherent and aligned with contemporary rTMS therapeutic guidelines [6]

Importantly, several trials suggest that cortical neuromodulation can translate into measurable changes in spinal circuitry. For example, rTMS has been shown to strengthen synaptic reciprocal Ia inhibition and improve impaired inhibitory control after traumatic spinal cord injury, consistent with increased descending facilitation of inhibitory interneurons [19]. In post-stroke upper-limb spasticity, combined 1-Hz rTMS and physiotherapy has been associated with reduced spinal excitability (e.g., H-reflex-related measures) alongside clinical MAS improvements, supporting a genuine neurophysiological substrate for symptom change beyond expectancy effects [39].

Beyond M1, additional network-level mechanisms may contribute to anti-spastic effects of these neuromodulatory interventions. Cerebellar iTBS, for instance, reduced upper-limb spasticity after subacute stroke, suggesting that modulating cerebello-thalamo-cortical loops can influence motor output and reflex regulation, potentially via changes in sensorimotor integration and descending control [31,51]. Similarly, in multiple sclerosis, iTBS-related spasticity improvement has been linked to modulation of resting-state functional connectivity between bilateral motor cortices, reinforcing the idea that rTMS acts on distributed motor networks rather than on a single cortical “hotspot” [24,30].

Another mechanistically salient point is the role of metaplasticity and state-dependence. Priming paradigms (e.g., pairing iTBS with high-definition transcranial direct current stimulation) aim to condition the local synaptic milieu and stabilize responses to subsequent stimulation. Although not all priming studies demonstrate superior spasticity outcomes, they consistently highlight that baseline cortical state and concurrent training influence clinical responsiveness [26]. More recently, brain state-dependent stimulation synchronized to sensorimotor μ-oscillations has shown feasibility and produced spasticity improvements comparable to conventional protocols, supporting the hypothesis that aligning stimulation to endogenous rhythms may reduce inter-individual variability [42].

Based on the assessment conducted using the GRADE system, the overall quality of evidence on the effectiveness of rTMS in reducing spasticity is moderate. This judgement is based on the review of all 26 RCTs included, most of which had a low risk of bias, but the heterogeneity of the stimulation protocols, the populations involved, and the assessment tools (e.g., MAS, Tardieu Scale, Visual Analogue Scale) has led to a certain degree of inconsistency between the results. In addition, in several studies, small sample sizes have led to a risk of imprecision. For secondary outcomes, such as improvement in motor function and quality of life, the certainty of the evidence was low or very low, due to the wide variability of results, the limited sample size, and a possible risk of publication bias. In many studies, indeed, functional outcomes have been considered as secondary measures, with heterogeneous instruments and often insufficient statistical power to detect clinically relevant differences. Overall, although these results suggest that rTMS may represent a promising rehabilitation strategy for the management of spasticity, further studies with larger samples and more standardised protocols are needed to consolidate the level of evidence and allow generalizability.

### 4.2. Safety Profile and Adverse Events

Overall, rTMS was well tolerated across the 26 included RCTs, with most studies reporting only mild and transient adverse events. The most frequently reported side effects were headache, scalp discomfort, transient skin irritation, fatigue, facial muscle twitching, and mild dizziness, typically occurring during or shortly after stimulation sessions and resolving spontaneously without intervention [18,22,25,27,33,39,42]. Less common adverse events included transient sleep disturbances, temporary worsening of spasticity, and mild autonomic symptoms, such as palpitations or nausea, which were sporadically reported and did not require treatment discontinuation [22,24,30,34]. Importantly, no persistent neurological deficit or long-term complication was described in any study.

Nevertheless, a single serious adverse event was reported in the RCT by Antczak et al. involving patients with hereditary spastic paraplegia [32], in which one participant experienced a generalized epileptic seizure leading to study withdrawal. According to the authors’ report, stimulation was delivered using a high-frequency protocol (10 Hz, 3000 pulses per session, double-cone coil) targeting the bilateral lower limb motor cortex. Although the stimulation parameters were within commonly accepted safety limits, the use of relatively high pulse numbers and bilateral stimulation may have increased cortical excitability. The original study did not report pre-existing epilepsy, structural brain lesions, or other major seizure risk factors in the affected participant, limiting the possibility to determine individual susceptibility. Notably, no other seizures were reported across the remaining trials, including studies using high-frequency stimulation, theta-burst protocols, or priming paradigms [21,24,26,30,42]. Furthermore, trials adhered to international safety recommendations regarding stimulation frequency, intensity, and inter-train intervals [6] and systematically screened participants for contraindications, such as previous epilepsy, metallic implants, or unstable medical conditions.

When considering incidence, mild adverse events were reported in 15–30% of participants, depending on the study and stimulation protocol, whereas serious adverse events occurred in less than 1% of treated individuals. Headache and scalp discomfort were more frequently observed with higher stimulation intensities and longer session durations, while facial twitching was mainly associated with stimulation of lower-limb motor representations using double-cone coils [22,27,34]. According to the system involved, neurological adverse events were the most frequently described adverse events and included headache, dizziness, transient fatigue, facial muscle twitching, and, in one isolated case, generalized seizure. These were followed by cutaneous and local adverse events, which mainly consisted of scalp discomfort, transient skin irritation, and localized pain at the stimulation site. Psychological and neuropsychiatric symptoms, such as transient anxiety, sleep disturbances, or mood changes, were rarely reported and were generally mild and self-limiting. Finally, autonomic and systemic symptoms, including nausea, palpitations, or transient malaise, were sporadically observed in a small number of participants only.

Taken together, the available evidence indicates that rTMS has a favourable safety profile for the treatment of spasticity when applied within established guidelines and after appropriate patient screening. Nevertheless, the occurrence of an isolated seizure highlights the importance of individualized risk assessment, cautious use of high-frequency and high-dose protocols, and standardized reporting of adverse events in future trials. Systematic documentation of safety outcomes, including event severity, timing, and potential protocol-related associations, is essential to optimize clinical risk–benefit balance.

Importantly, adverse event reporting was not standardized across the included studies. Most trials relied on spontaneous self-reporting by participants, investigator-led interviews, or routine clinical observation during and after stimulation sessions. Only a minority of studies explicitly described the use of structured or standardized adverse event assessment tools, and none consistently applied internationally validated safety questionnaires or formal grading systems. Consequently, frequency and severity of adverse events may have been underestimated, particularly for mild or delayed symptoms.

### 4.3. Mechanistic Rationale for Disease-Specific Stimulation Strategies

The apparent divergence in optimal stimulation paradigms across different neuromotor disorders can be more precisely interpreted considering disease-specific neural circuit pathology. In post-stroke spasticity, cortical lesions frequently disrupt corticospinal output and alter interhemispheric balance, resulting in excessive transcallosal inhibition from the contralesional hemisphere and compensatory hyperexcitability of brainstem descending pathways, particularly the reticulospinal tract [4,50]. This maladaptive interhemispheric inhibition contributes to impaired voluntary control and increased stretch reflex gain. Therefore, low-frequency (≤1 Hz) inhibitory stimulation applied to the contralesional motor cortex may reduce excessive interhemispheric inhibition, rebalance cortical excitability, and indirectly decrease downstream spinal hyperexcitability [6,25,40].

Beyond theoretical models of network reorganization, several neurophysiological studies support the disease-specific selection of stimulation frequency and cortical targets. In post-stroke patients, low-frequency rTMS applied to the contralesional motor cortex has been shown to reduce motor evoked potential (MEP) amplitudes and to prolong cortical silent period (CSP) duration, reflecting enhanced intracortical inhibition and reduced maladaptive transcallosal drive. These changes are frequently accompanied by normalization of interhemispheric MEP asymmetry and improvements in reciprocal inhibition at the spinal level, which correlate with reductions in spasticity scores [52,53].

In contrast, in incomplete spinal cord injury, the primary mechanism underlying spasticity involves loss of descending supraspinal inhibitory control combined with intrinsic spinal hyperexcitability and altered interneuronal circuitry [19,22]. Because residual cortico-spinal fibers may remain anatomically preserved but functionally underactive, high-frequency or theta-burst excitatory stimulation targeting the ipsilesional motor cortex may enhance descending drive, strengthen synaptic transmission, and facilitate restoration of deputed inhibitory spinal circuits, such as reciprocal Ia inhibition [19,21,22].

Similarly, in multiple sclerosis, demyelination and network disconnection affect distributed motor pathways, often producing fluctuating conduction failure rather than complete structural interruption [24,30]. In this context, excitatory protocols (e.g., HF-rTMS or iTBS) may transiently enhance conduction efficiency and network synchronization within motor circuits, potentially explaining the reported improvements in both spasticity and fatigue [24].

In incomplete spinal cord injury and multiple sclerosis, high-frequency rTMS and iTBS have been associated with increased MEP amplitudes, reduced motor thresholds, and enhanced short-interval intracortical facilitation, indicating strengthened cortico-spinal excitability and improved synaptic transmission. These excitatory effects appear to facilitate residual descending pathways and restore inhibitory control over spinal reflex circuits, providing a physiological rationale for the preferential use of facilitatory protocols in these conditions [54,55,56,57]. Furthermore, studies employing paired-pulse TMS and combined TMS–EEG paradigms have demonstrated that different stimulation targets modulate distinct cortical and subcortical networks. For example, cerebellar or premotor stimulation influences sensorimotor integration and corticocortical connectivity, whereas primary motor cortex stimulation primarily affects cortico-spinal output. These target-specific neurophysiological signatures further justify the use of different stimulation sites according to disease-specific circuit dysfunction [58,59,60].

Thus, the selection of inhibitory versus excitatory stimulation strategies appears mechanistically coherent when interpreted according to the specific pathophysiological substrate of each disorder, rather than representing contradictory therapeutic approaches.

### 4.4. Comparison with Previous Systematic Reviews and Incremental Contribution

The findings of the present review are broadly consistent with previous systematic reviews and meta-analyses indicating that rTMS may reduce spasticity after upper motor neuron injury, particularly in stroke populations [44]. Prior pooled analyses have generally reported modest results except for statistically significant reductions in MAS scores, especially following multi-session stimulation protocols combined with rehabilitation.

However, most previous reviews either focused exclusively on post-stroke spasticity or combined heterogeneous neurological conditions without structured stratification by diagnosis, stimulation parameters, and certainty of evidence [44,45]. In contrast, the present review systematically grouped results by underlying pathology (stroke, spinal cord injury, multiple sclerosis, cerebral palsy, hereditary spastic paraplegia), analyzed protocol-specific trends, and integrated a formal risk-of-bias and GRADE certainty assessment across 26 RCTs. Furthermore, unlike earlier meta-analyses primarily centred on pooled effect sizes, this review provides a mechanistic interpretation linking stimulation paradigms to disease-specific neural circuit alterations and offers a structured analysis of clinical and methodological heterogeneity. Therefore, the incremental contribution of this study lies in its updated inclusion of recent RCTs (including state-dependent and priming paradigms), cross-disease comparative framework, and integration of pathophysiological interpretation with evidence grading, thereby offering a more nuanced and clinically oriented synthesis of the current evidence base.

### 4.5. Translational Considerations

Implications for clinical practice are relevant: the available RCT evidence suggests indeed that rTMS may be best considered as an adjunct to conventional rehabilitation rather than a stand-alone intervention. Namely, clinically meaningful reductions in spasticity are more frequently reported when stimulation is delivered daily over multi-session courses (often ≥10 sessions) and paired with task-oriented physiotherapy. Benefits appear more robust for spasticity outcomes than for broader endpoints, such as quality of life, which remain basically inconsistent. Where follow-up was assessed, improvements may persist for weeks, although they tend to attenuate after discontinuation, supporting the need for adequate maintenance strategies.

According to the GRADE framework (Table 3), the overall certainty of evidence for spasticity reduction remains moderate, reflecting generally low risk of bias but relevant limitations related to inconsistency/imprecision. In clinical terms, this rating indicates that further well-designed trials are likely to have an important impact on the estimated effect and may change current conclusions. Therefore, the available evidence is sufficient to support cautious initial clinical application of rTMS as an adjunct to standard rehabilitation in selected patients, particularly in post-stroke and incomplete spinal cord injury populations. However, it does not yet justify routine or universal implementation as a standard-of-care intervention. More adequately powered, multicentre RCTs with standardized protocols, harmonized outcome measures, and longer follow-up are required to confirm efficacy, define optimal treatment parameters, and establish long-term cost–benefit profiles.

Taken together, these mechanistic insights translate into several practical considerations. First, rTMS is more likely to be effective in patients with preserved corticospinal connectivity or at least demonstrable cortico-spinal excitability, because the intervention relies on descending pathways to influence spinal inhibitory networks. Second, targeting and dosing should match the pathophysiological hypothesis: contralesional inhibitory stimulation to address maladaptive interhemispheric inhibition in stroke, versus excitatory stimulation to augment residual descending drive in incomplete spinal cord injury and other conditions. Third, pairing stimulation with goal-directed, task-specific training may capitalize on Hebbian mechanisms and consolidate plastic changes, potentially extending benefit duration. Finally, individualized approaches (e.g., neuronavigation, state-dependent stimulation, high-density EEG) may help identify responders and optimize resource utilization in “real world” rehabilitation settings [61].

### 4.6. Heterogeneity, Limitations, and Implications for Interpretation

A major limitation of the present review is the substantial clinical and methodological heterogeneity across the included studies, which significantly affects the interpretation of results and limits the comparability of findings. This heterogeneity arises from several interrelated sources, including pathophysiological differences between disease groups, variability in stimulation protocols, heterogeneity in outcome assessment tools, and differences in concomitant interventions.

First, important pathophysiological differences exist among the neuromotor disorders included in this review. Post-stroke spasticity, as known, is primarily related to disruption of cortico-spinal and interhemispheric inhibitory mechanisms, leading to maladaptive cortical reorganization and enhanced reticulo-spinal drive [4,50]. In contrast, spasticity in spinal cord injury is mainly associated with loss of supraspinal inhibition and intrinsic spinal hyperexcitability, including impaired reciprocal inhibition and altered interneuronal circuitry [19,22]. In multiple sclerosis, demyelination, neuroinflammation, and diffuse network dysfunction contribute to spasticity through both central and peripheral mechanisms [24,30]. Finally, cerebral palsy and hereditary spastic paraplegia represent developmental and genetic disorders, respectively, characterized by long-standing structural and functional alterations of motor pathways [20,32,37]. These distinct neurobiological substrates likely influence responsiveness to neuromodulation and partly explain the variability in treatment effects observed across conditions.

Second, there was marked heterogeneity in rTMS parameters across studies. Protocols differed substantially in stimulation frequency (ranging from inhibitory 0.5–1 Hz to excitatory 5–20 Hz and theta-burst stimulation), cortical targets (ipsilesional vs. contralesional M1, premotor cortex, cerebellum), coil type (figure-of-eight vs. double-cone), intensity relative to motor threshold, total pulse number, and treatment duration [6,22,25,31,42]. Such variability reflects not only different pathophysiological hypotheses and therapeutic strategies but also limits direct comparisons between trials. Previous methodological reviews have emphasized that rTMS effects are highly dependent on dose, target, and stimulation pattern, and that small variations may result in divergent neurophysiological and clinical outcomes [6,44,46]. Consequently, the absence of standardized protocols contributes substantially to inter-study variability.

Third, outcome measurements differed considerably among studies. Although MAS was the most frequently used primary outcome, several trials employed additional or alternative measures, including the Tardieu Scale, Spinal Cord Assessment Tool, functional motor scales, gait parameters, and patient-reported outcomes [23,25,30,34]. MAS, while widely used, has limited sensitivity to change and is influenced by some biomechanical factors, such as contractures and soft-tissue stiffness [50]. Moreover, it only captures resistance to passive movement and does not fully reflect functional or patient-perceived impact of spasticity. Therefore, heterogeneous outcome instruments and assessment time points contribute to measurement inconsistency and hinder quantitative synthesis.

Fourth, substantial variation was observed in the use of combined interventions. Many studies paired rTMS with physiotherapy, occupational therapy, repetitive facilitation exercises, constraint-induced movement therapy, or robotic gait training, whereas others applied rTMS as a stand-alone intervention [23,25,27,28,38,39]. Such combinations may enhance neuroplastic effects through activity-dependent mechanisms and Hebbian learning principles [46,61]. However, they also introduce confounding effects, making it difficult to disentangle the specific contribution of neuromodulation from that of rehabilitation. Differences in intensity, duration, and content of concomitant therapies further complicate cross-study comparisons.

The combined impact of these heterogeneous factors has important implications for the interpretation of results. First, it reduces internal validity and increases the risk that observed effects reflect context-specific interactions between disease mechanisms, stimulation parameters, and rehabilitation strategies rather than generalizable treatment effects. Second, it limits external validity, as findings obtained in narrowly defined populations using specific protocols may not be transferable to other clinical settings. Third, heterogeneity undermines the feasibility and reliability of meta-analytic approaches, as pooling results across markedly different interventions and populations may yield misleading estimates of treatment efficacy [14,44]. Furthermore, heterogeneity contributes to variability in treatment responsiveness at the individual level. Several studies, indeed, suggest that baseline cortico-spinal integrity, lesion location, disease duration, and cortical excitability state may influence responsiveness to rTMS [26,42,46]. The lack of systematic stratification according to these factors in most trials may have masked clinically relevant subgroup effects and contributed to inconsistent findings.

Although the main sources of heterogeneity have been identified, the lack of systematic integration between clinical outcomes and neurophysiological biomarkers represents a major barrier to protocol harmonization. Most trials measured spasticity without parallel assessment of cortical excitability, intracortical inhibition/facilitation, or cortico-spinal conduction. As a result, it remains difficult to determine whether similar clinical improvements arise from comparable neural mechanisms or from distinct compensatory processes, eventually limiting the development of unified stimulation strategies.

Lastly, additional limitations that must be considered when interpreting these results are: (i) the search strategy was focused specifically on the terms “spasticity” and “muscle spasticity”; although this choice ensured a high degree of specificity in the selection of studies, it is possible that some clinical trials focusing on muscle hypertonia but indexed with alternative terminology (e.g., “hypertonia”, “muscle tone”, “rigidity”) or mentioning spasticity only in the full text and not in the abstract were not identified; (ii) the restriction to articles in English may have introduced a linguistic selection bias; (iii) the relatively small sample sizes and often short follow-up periods limit the ability to accurately estimate the magnitude and duration of clinical effects; (iv) some studies have methodological shortcomings (e.g., incomplete description of randomisation), which, while not invalidating the results, contribute to lowering the overall level of certainty of the evidence according to the GRADE system; (v) most trials did not adopt standardized adverse event recording instruments and used variable, often informal, methods for documenting side effects, thereby limiting the comparability and reliability of safety data.

Taken together, these considerations indicate that the current evidence base should be interpreted cautiously. While most studies report favourable effects of rTMS on spasticity, the magnitude, durability, and clinical relevance of these effects remain difficult to quantify. Future RCTs should prioritize standardized reporting of stimulation parameters, harmonized outcome sets, and detailed documentation of co-interventions, in line with current methodological recommendations [6,14,44]. Moreover, stratification by disease subtype, lesion characteristics, and baseline neurophysiological markers may help reduce heterogeneity and improve the interpretability and reproducibility of findings.

To facilitate the development of consensus-based protocols, future research should adopt coordinated methodological frameworks. First of all, stimulation parameters should be systematically explored using dose–response designs, varying frequency, intensity, and pulse number within the same disease population while monitoring neurophysiological markers, such as MEP amplitude, CSP duration, and paired-pulse indices. Also, multicentre trials should agree on a limited set of “core” stimulation protocols for each major disease category, based on preliminary physiological and clinical evidence.

## 5. Conclusions

This systematic review indicates that rTMS may represent a relatively safe and potentially effective adjunctive intervention for the management of spasticity in several neuromotor disorders, particularly in post-stroke and incomplete spinal cord injury populations. Moderate-certainty evidence supports its ability to reduce upper and lower limb spasticity when delivered in multi-session protocols and combined with structured rehabilitation. From a pure clinical perspective, however, rTMS should currently be considered as a complementary tool within multidisciplinary neurorehabilitation programs rather than a stand-alone therapy. Its use may be most appropriate in carefully selected patients and specialized settings, where individualized targeting and close monitoring are available. Nevertheless, substantial heterogeneity in stimulation parameters, outcome measures, and study designs currently limits the generalizability of the available findings and precludes strong recommendations for routine clinical implementation.

Further large-scale, multicenter RCTs with standardized protocols, harmonized outcome sets, integrated neurophysiological biomarkers, and longer follow-up periods are required to establish optimal treatment strategies and long-term effectiveness. Future research should also prioritize protocol harmonization, biomarker-guided patient stratification, and systematic evaluation of combined neuromodulation–rehabilitation approaches to support the development of evidence-based clinical guidelines for rTMS in spasticity. Progress in this field will mainly depend on the integration of neurophysiological biomarkers with standardized clinical outcomes and coordinated multicenter trial designs, enabling the rational selection of stimulation frequency and targets and, at the same time, supporting the development of consensus-based therapeutic protocols.

## Figures and Tables

**Table 1:** Summary of the studies included in this systematic review.

N.	Authors (Year)	Disease Model	Type of Study	Sample Size	Intervention	Control	Outcome	Follow Up Time Points	Main Findings
1	Gottlieb et al. (2021) [18]	Post-stroke patients with upper limb spasticity	RCT, double-blind	28 (14 rTMS, 14 sham)	1 Hz inhibitory rTMS (LF-rTMS) on the contralateral M1: 1200 pulses/session, 10 sessions over 12 days, + occupational therapy and electrostimulation	Sham-rTMS + occupational therapy and electrostimulation	Modified Ashworth Scale (MAS), Fugl-Meyer Assessment (FMA)	Baseline; 3–4 days before and after treatment	Significant reduction in spasticity (MAS) only in the rTMS group; motor improvement (FMA) in both groups.
2	Nardone et al. (2014) [19]	Patients with spasticity due to incomplete traumatic SCI	RCT crossover, double-blind	9 patients with SCI (8 received real rTMS), 8 healthy controls	5 consecutive sessions of rTMS (20 Hz, 1600 total pulses/day, contralateral M1, 90% of the motor threshold MT of the biceps brachii)	5 initially sham, 4 of these crossed over to real rTMS	MAS	Baseline; 1 week post treatment	Significant reduction in spasticity (MAS) and improvement in reciprocal inhibition only after real rTMS. No change after sham. Effects partially persistent at 1 week.
3	Bastani et al. (2023) [20]	Hereditary Spastic Paraplegia	RCT, single-blind	8 (4 rTMS, 4 sham)	5 consecutive sessions of rTMS 5 Hz, 1500 pulses per session, 90% resting motor threshold	Sham rTMS (coil placed perpendicular to the scalp)	Modified Ashworth Scale (MAS), 10-m Walking Test (10MWT), Fugl-Meyer Assessment for Lower Extremity (FMA-LE)	Baseline; 1 month after treatment	rTMS significantly reduced spasticity (MAS) compared to the sham group
4	Nardone et al. (2017) [21]	Incomplete cervical or thoracic spinal cord injury	RCT, double-blind, crossover	10 patients (7 M, 3 F, aged 24–65)	iTBS on the dominant hemisphere M1 (600 stimuli/day for 10 days)	Sham iTBS	MAS (Modified Ashworth Scale), SCAT	Baseline; 1 week and 4 weeks after treatment	Significantly reduced MAS and SCAT; effects observed up to 1-week post-treatment. No effect with sham.
5	Kumru et al. (2010) [22]	Spasticity in patients with incomplete SCI	RCT, double-blind, crossover	15 (14 received active rTMS, 6 crossover)	rTMS 20 Hz, 90% of the resting motor threshold of the biceps brachii, 5 sessions/day (20 trains × 40 pulses; 1600 total pulses/day, for 5 days)	Sham rTMS (7 patients)	MAS, VAS, SCAT	Baseline; 1 week after treatment	Significant reduction in spasticity after active rTMS (MAS, VAS, SCAT), effect maintained at 1 week; no neurophysiological effects observed; no improvement in the sham group.
6	Rastgoo et al. (2016) [23]	Stroke (chronic post-stroke patients)	RCT Cross-over	20 (14 crossover)	5 consecutive daily sessions of rTMS (1 Hz, 1000 pulses, 90% anterior tibial motor threshold) on the motor area of the unaffected hemisphere	Sham rTMS with audio coil (no magnetic stimulation)	MAS, LE-FMA (motor function), TUG (gait)	Baseline; 1 week after treatment	Significant improvement in spasticity (MAS) and motor function (LE-FMA) after active rTMS, maintained at 1 week. No effect with sham.
7	Korzhova et al. (2019) [24]	Secondary progressive multiple sclerosis (SPMS)	RCT	34 patients (12 HF-rTMS, 12 iTBS, 10 sham)	- HF-rTMS: 20 Hz, 10 sessions over 2 weeks, 1600 stimuli/session - iTBS: 10 sessions over 2 weeks, 1200 stimuli/session	Sham stimulation (coil inactive at 1 m)	MAS, MFIS (Modified Fatigue Impact Scale)	Baseline; 12 weeks after treatment	Significant reduction in spasticity (MAS) with HF-rTMS and iTBS (not in the sham group) - Significant reduction in pain and fatigue with HF-rTMS, not with iTBS
8	Kuzu et al. (2021) [25]	Chronic ischemic stroke with upper limb spasticity	RCT, double-blind	20 patients	- rTMS: 1 Hz, 1200 pulses, 90% resting motor threshold, 10 sessions - cTBS: 600 pulses, 80% active motor threshold, 10 sessions - Both on the non-lesional hemisphere + physiotherapy	Sham cTBS + physiotherapy	- Modified Ashworth Scale (MAS) - Fugl-Meyer Assessment (FMA) - Functional Independence Measure (FIM) - Motor Activity Log-28 (MAL-28) - Brunnstrom scale	Baseline; 4 weeks after treatment	- rTMS and cTBS significantly improve motor function (FMA) and independence (FIM, MAL-28) compared to the sham group. - rTMS significantly reduces spasticity in elbow flexors, pronators, wrist flexors, and fingers. - cTBS improves spasticity only in elbow and wrist flexors. - No improvement in the sham group. - Improvements maintained at 4 weeks.
9	Bian et al. (2024) [26]	Subacute stroke (patients with upper limb hemiparesis)	RCT	66 (60 complete)	15 sessions (3 weeks) of: (1) cathodal HD-tDCS priming (20 min on the injured M1, 1.5 mA) followed by iTBS (ipsilesional, 600 pulses, 80% AMT); (2) iTBS without priming; both associated with standard rehabilitation	iTBS without HD-tDCS and sham HD-tDCS + sham iTBS	Fugl-Meyer Assessment—Upper Extremity (FMA-UE), Motricity Index—Upper Extremity (MI-UE), Modified Barthel Index (MBI), FTHUE-HK, MAS	Baseline; end of treatment (3 weeks)	The tDCS + iTBS group showed significantly greater improvements in FMA-UE, MI-UE, and MBI compared to the sham group. No significant improvement in FTHUE-HK and MAS. No significant benefit for non-priming iTBS.
10	Kumru et al. (2016) [27]	Incomplete motor spinal cord injury (<6 months)	RCT, double-blind	31 (15 rTMS, 16 sham)	20 Hz, 1800 pulses/session, 20 sessions total (1/day × 4 weeks), double cone coil on the vertex	Sham rTMS with coil disconnected	MAS (Modified Ashworth Scale), UEMS, LEMS, 10MWT	Baseline; 4 weeks after treatment	MAS: no significant change. LEMS and UEMS improved significantly in the rTMS group compared to sham. More subjects in the rTMS group were able to perform the 10MWT at follow-up (71.4% vs. 40%), but the difference was not significant. No serious side effects.
11	Barros Galvão et al. (2014) [28]	Chronic stroke (patients with upper limb spasticity)	RCT, double-blind	20 (10 rTMS + PT, 10 sham + PT)	10 rTMS sessions (1 Hz, 1500 pulses, 90% resting motor threshold, unaffected hemisphere) + physiotherapy	Sham rTMS + physiotherapy	Primary: MAS (Modified Ashworth Scale); Secondary: UE-FMA, FIM, wrist ROM, SS-QOL	Baseline; after treatment	The rTMS group showed significant reductions in spasticity (MAS −0.9 post-intervention; −0.6 follow-up), with clinically relevant improvements in 90% post and 55.5% follow-up. No significant differences between groups on other outcomes.
12	Abdelkader et al. (2024) [29]	Ischemic stroke in the subacute phase (within 6 months), with hemiparesis	RCT with 4 arms, single-blind	55 patients (15 + 15 + 15 + 10)	- A: 5 Hz on the affected hemisphere—B: 1 Hz on the healthy hemisphere—C: bilateral (1 Hz on the healthy hemisphere and 5 Hz on the affected hemisphere, every other day), 10 sessions in 2 weeks	D (sham, 10 patients)	MAS, MRC, Brunnstrom stages, Barthel Index, 10MWT	Baseline; end of treatment (2 weeks)	All active groups showed significant improvements on MAS and MRC compared to the sham group, with superiority of the bilateral group (C). No adverse events reported.
13	Boutière et al. (2017) [30]	Multiple sclerosis (patients with spasticity affecting a lower limb, EDSS 4–7, MAS ≥ 2)	RCT	17 (9 real iTBS, 8 sham iTBS)	iTBS on the lower limb area of the primary motor cortex (13 sessions over 3 weeks, 600 stimuli/session)	iTBS sham + same rehabilitation	Modified Ashworth Scale (MAS), Visual Analogue Scale (VAS)	Baseline; 3 weeks and 5 weeks after treatment	Real iTBS + rehabilitation reduced spasticity more significantly; correlation between improvement and change in functional connectivity; transient effect disappeared at W5
14	Chen et al. (2021) [31]	Post-stroke upper limb spasticity in the subacute phase	RCT, double-blind	32 (16 iTBS, 16 sham)	Intermittent cerebellar iTBS on the ipsilesional side, 10 sessions (600 pulses, 80% aMT, 2 weeks)	Sham stimulation	MAS, MTS, Barthel Index (secondary)	Baseline; post-treatment (10 sessions); no long-term follow-up	Cerebellar iTBS significantly reduced spasticity (MAS, MTS) compared to the sham group. Improvements were also seen in the Barthel Index in both groups. No adverse events were recorded.
15	Antczak et al. (2019) [32]	Hereditary spastic paraplegia (pure, complicated forms and Adreno-myeloneuropathy); adults	RCT crossover	15 (14 complete)	5 consecutive sessions of bilateral rTMS on the primary motor area of the lower limbs, 10 Hz, 3000 pulses/session (1500 per hemisphere), intensity at 90% of RMT or AMT, double cone coil	Sham with coil rotated 90°	10MWT, TUG, (MAS), muscle strength with dynamometer, MEP, CMCT, CSP	Baseline; 2 weeks after treatment	↑ proximal and distal lower limb strength; ↓ proximal muscle spasticity; no effect on walking (10MWT, TUG); no effect after sham; one epileptic seizure in one patient (drop-out)
16	Şengül et al. (2023) [33]	Stroke (patients with moderate to severe spastic paresis of the upper limb, chronic > 12 months)	RCT, double-blind	37 (12 LFrTMS, 12 HFrTMS, 13 Sham)	A single session: - LFrTMS (1 Hz) - HFrTMS (10 Hz) on the contralateral dorsal premotor cortex	Sham rTMS (sham stimulation)	Modified Ashworth Scale (MAS)	Baseline; immediate post (single session)	Only HFrTMS showed a significant reduction in MAS in the group itself but no significant difference compared to Sham.
17	Gharooni et al. (2018) [34]	Incomplete cervical SCI, 10 participants	RCT, cross-over, single-blind	10	iTBS (intermittent theta-burst stimulation) administered to the primary motor cortex, 10 sessions over 2 weeks, 600 pulses/session at 80% RMT	iTBS sham (coil rotated 90° to avoid brain stimulation)	MAS, LASIS, VAS-S, ASIA motor (UEMS/LEMS),	Baseline; 2 weeks after treatment	iTBS reduced upper limb spasticity according to MAS, but without clinically meaningful perceived improvements (VAS-S, LASIS). No significant effect on pain or motor function.
18	Terreaux et al. (2014) [35]	patients with spasticity due to post-stroke hemiparesis (4) and post-surgery for meningioma (1)	RCT, crossover, double-blind	5 patients	rTMS on lesioned premotor cortex: - 1 Hz at 90% motor threshold - 10 Hz at 100% motor threshold - 1000 pulses/day for 5 days	sham stimulation	- Modified Ashworth Scale - Antagonist recruitment (scale 1–4) - Fugl-Meyer scale - Gait analysis (kinematic and temporal)	Baseline; 4 days and 21 days after treatment	- No significant changes with placebo and - No clinical changes on Ashworth
19	Chen et al. (2019) [36]	Chronic unilateral stroke	RCT single-blind	22 (11 iTBS, 11 sham)	iTBS on the primary motor area ipsilesional, 10 sessions in 2 weeks (5/week), 600 pulses/session	Sham iTBS, same setting	MAS, FMA-UE, ARAT (grasp, grip, pinch, gross), BBT, MAL (AOU e QOM)	Baseline; immediate post (no follow up)	iTBS significantly reduced spasticity (MAS) and improved fine motor function (ARAT, FMA-UE, BBT); no significant difference for MAL
20	Valle et al. (2007) [37]	Cerebral palsy (quadriplegic spasticity, average age 9.1 years)	RCT double-blind	17 patients (5 Hz: 5; 1 Hz: 6; Sham: 6)	5 consecutive days, 5 Hz rTMS, 1500 pulses/day, 90% of motor threshold, primary cortical motor stimulation	Sham coil with identical parameters (5 Hz or 1 Hz)	Ashworth Scale (MAS), Range of Motion (ROM) with goniometer,	Baseline; 2 h after the last session	Significant improvement in ROM (wrist flexion and extension, elbow flexion) in the 5 Hz group. No significant effect on MAS.
21	Etoh et al. (2013) [38]	Chronic stroke (patients with upper limb hemiplegia)	RCT, double-blind, crossover	18 patients	1-Hz rTMS on the unaffected motor hemisphere for 4 min (240 pulses) + repetitive facilitation exercises (RFE) for 40 min	sham rTMS (5 cm posterior area)	Fugl-Meyer Assessment (FMA), Action Research Arm Test (ARAT), Simple Test for Evaluating Hand Function (STEF), MAS	Baseline; 4 weeks after treatment	Significant improvement in motor function (ARAT, FMA, STEF) during true rTMS compared to sham; no significant change in spasticity (MAS)
22	Costa dos Santos et al. (2019) [39]	Post-stroke upper limb spasticity (chronic post-stroke patients)	RCT, double-blind	20 (10 rTMS + PT, 10 sham + PT)	1 Hz rTMS on the unaffected hemisphere (1500 pulses, 90% motor threshold at rest) for 10 sessions, combined with physiotherapy	Sham rTMS + physiotherapy	Cortical excitability (MSO), spasticity (Modified Ashworth Scale—MAS)	Baseline; 4 weeks after treatment	Increased cortical excitability in the unaffected hemisphere (↓ MSO), ↓ spasticity (↓ MAS) from the 6th treatment. Greater effects in the rTMS + PT group.
23	Chervyakov et al. (2018) [40]	Patients with unilateral ischaemic stroke	RCT, 4-arm	42 patients (completed), initially 64	rTMS navigated in 3 modes: 1) Low frequency 1 Hz unaffected hemisphere, 2) High frequency 10 Hz affected hemisphere, 3) Sequential combination of 1–10 Hz	Sham rTMS (N = 10)	Fugl-Meyer Assessment (FM), Modified Ashworth Scale (MAS), Barthel Index (BI)	Baseline; end of treatment	Significant improvements in FM for all 3 rTMS groups. MAS and BI improved in the 1 Hz and 10 Hz groups, but not in the combined group. No improvement in the sham group.
24	Mufti et al. (2025) [41]	Hemiplegic cerebral palsy (ages 5–18)	RCT, double-blind	40 (20 rTMS, 20 sham)	10 sessions over 4 weeks: 6 Hz priming (600 pulses) + 1 Hz rTMS (600 pulses) on the contralateral hemisphere; combined with 10 sessions of mCIMT.	Sham rTMS + mCIMT	MAS	Baseline; immediate post treatment (no follow-up)	reduction in spasticity (MAS) in the rTMS group. was observed. No significant improvement was observed in the sham group.
25	Mahmoud et al. (2024) [42]	Patients with chronic post-stroke spasticity (≥6 months)	RCT, double-blind	30 patients (15 per group)	100 Hz (triplets) EEG-guided rTMS on the ipsilesional M1, synchronised to the negative phase of the μ-oscillation (average frequency 0.33 Hz, 1200 pulses)	1 Hz rTMS on contralateral M1 (standard), 1200 pulses at 115% RMT	Modified Ashworth Scale (MAS), Stretch Reflex Torque (dynamometer)	Baseline; immediate post-treatment; 3 months after treatment	Significant improvement in spasticity (MAS and stretch reflex torque) and motor function in both groups, with no significant differences between the two protocols. Effects maintained at 3 months.
26	Mahgoub et al. (2021) [43]	Spastic hemiplegic cerebral palsy	RCT	30 (15 rTMS + 15 control)	High frequency rTMS (10 Hz, 1500 pulses/day × 20 days) + standard physical therapy	Standard physical therapy only (1 h/day, 5 days/week for 4 weeks)	Modified Ashworth Scale (MAS), 3D gait analysis (Q-Trac System)	Baseline; 3 months after treatment	Significant reduction in spasticity and improvement in gait parameters (speed, stride length, ankle angle) in the rTMS group compared to the control group.

Legend (in alphabetical order): 10MWT, 10-Meter Walking Test; AMT, active motor threshold; ARAT, Action Research Arm Test; ASIA, American Spinal Injury Association; BBT, Box and Block Test; BI, Barthel Index; EDSS, Expanded Disability Status Scale; FIM, Functional Independence Measure; FMA, Fugl-Meyer Assessment; FMA-LE, Fugl-Meyer Assessment—Lower Extremity; FMA-UE, Fugl-Meyer Assessment—Upper Extremity; FTHUE-HK, Functional Test for the Hemiplegic Upper Extremity—Hong Kong version; HF-rTMS, high-frequency repetitive transcranial magnetic stimulation; iTBS, intermittent theta-burst stimulation; LASIS, Leeds Adult Spasticity Impact Scale; LEMS, Lower Extremity Motor Score; MAL, Motor Activity Log; MAL-28, Motor Activity Log-28; MAS, Modified Ashworth Scale; MBI, Modified Barthel Index; MFIS, Modified Fatigue Impact Scale; MI-UE, Motricity Index—Upper Extremity; MRC, Medical Research Council muscle strength scale; MSO, maximum stimulator output; PT, physiotherapy; Q-Trac, Q-Trac 3D gait analysis system; RFE, repetitive facilitation exercises; RMT, resting motor threshold; ROM, range of motion; SCAT, Spinal Cord Assessment Tool for Spastic Reflexes; SCI, spinal cord injury, SS-QOL, Stroke-Specific Quality of Life scale; STEF, Simple Test for Evaluating Hand Function; TUG, Timed Up and Go test; VAS, Visual Analogue Scale; VAS-S, Visual Analogue Scale for spasticity; ↑, increased/enhanced; ↓, decreased/reduced.

**Table 2:** Summary of the risk of bias assessments for each study.

N.	Study	Randomization	Deviations Intervention	Missing Data	Outcome Measurement	Selection Results	Overall Risk
1	Gottlieb et al., (2021) [18]	Low	Low	Low	Some concerns	Low	Some concerns
2	Nardone et al., (2014) [19]	Some concerns	Low	Low	Some concerns	Low	Some concerns
3	Bastani et al., (2023) [20]	Low	Low	Low	Low	Low	Low
4	Nardone et al. (2017) [21]	Low	Low	Low	Low	Low	Low
5	Kumru et al. (2010) [22]	Low	Low	Low	Low	Low	Low
6	Rastgoo et al., (2016) [23]	Some concerns	High	Low	Some concerns	Some concerns	High
7	Korzhova et al., (2019) [24]	Low	Some concerns	Low	Some concerns	Some concerns	Some concerns
8	Kuzu et al. (2021) [25]	Low	Low	Low	Low	Low	Low
9	Bian et al., (2024) [26]	Low	Low	Low	Low	Low	Low
10	Kumru et al., (2016) [27]	Low	Low	Low	Low	Low	Low
11	Barros Galvão et al., (2014) [28]	Low	Low	Low	Some concerns	Low	Some concerns
12	Abdelkader et al., (2024) [29]	Low	Low	Low	Some concerns	Low	Some concerns
13	Boutière et al., 2017 [30]	Low	Low	Low	Some concerns	Low	Some concerns
14	Chen et al. (2021) [31]	Low	Low	Low	Some concerns	Low	Some concerns
15	Antczak et al., (2019) [32]	Low	Low	Low	Some concerns	Low	Some concerns
16	Şengül et al. (2023) [33]	Low	Low	Low	Some concerns	Low	Some concerns
17	Gharooni et al., (2018) [34]	Some concerns	Some concerns	Some concerns	Some concerns	Low	Some concerns
18	Terreaux et al., (2014) [35]	High	Low	Low	Some concerns	High	High
19	Chen et al., (2019) [36]	Low	Low	Low	Low	Low	Low
20	Valle et al., (2007) [37]	Low	Low	Low	Low	Some concerns	Some concerns
21	Etoh et al., (2013) [38]	Low	Low	Low	Low	Some concerns	Some concerns
22	Costa dos Santos et al., (2019) [39]	Low	Low	Low	Low	Low	Low
23	Chervyakov et al., (2018) [40]	Low	Some concerns	High	Low	Low	High
24	Mufti et al., (2025) [41]	Low	Low	Some concerns	Low	Low	Low
25	Mahmoud et al., (2024) [42]	Low	Low	Low	Low	Low	Low
26	Mahgoub et al., (2021) [43]	Low	Low	Low	Some concerns	Low	Some concerns

**Table 3:** Summary of GRADE assessment for key outcomes.

Outcome	No. of Studies (RCTs)	Participants (Approximately)	Risk of Bias	Inconsistency	Imprecision	Publication Bias	Certainty of Evidence (GRADE)	Main Reasons for Downgrading
Upper limb spasticity	14	~500	Low–Moderate	Moderate	Moderate	Undetected	Moderate	Protocol heterogeneity, small samples
Lower limb spasticity	12	~350	Low–Moderate	Moderate	Moderate	Undetected	Moderate	Clinical heterogeneity, imprecision
Safety (adverse events)	26	~800	Low	Low	Low	Undetected	Moderate	Rare serious events, limited reporting
Motor function	15	~400	Moderate	High	High	Possible	Low	Inconsistent results, small samples
Quality of life	6	~180	Moderate	High	High	Possible	Very Low	Few studies, heterogeneous tools

## Data Availability

All relevant data are included in the manuscript and its Supplementary Materials.

## Supplementary Materials

The following supporting information can be downloaded at: https://www.mdpi.com/article/10.3390/jcm15051932/s1, Table S1: PRISMA 2020 Checklist [14].
